# In myotonic dystrophy type 1 reduced FDG-uptake on FDG-PET is most severe in Brodmann area 8

**DOI:** 10.1186/s12883-016-0630-3

**Published:** 2016-07-13

**Authors:** Dimitri Renard, Laurent Collombier, Christel Castelli, Jean-Pierre Pouget, Pierre-Olivier Kotzki, Vincent Boudousq

**Affiliations:** Department of Neurology, CHU Nîmes, Hôpital Caremeau, Place du Pr Debré, 30029 Nîmes Cedex 4, France; Department of Nuclear Medicine, CHU Nîmes, Hôpital Caremeau, Place du Pr Debré, 30029 Nîmes Cedex 4, France; Laboratoire de Biostatistique, Epidémiologie clinique, Santé Publique et Information, Médicale (BESPIM), CHU Nîmes, Hôpital Caremeau, Place du Pr Debré, 30029 Nîmes Cedex 4, France

## Abstract

**Background:**

In myotonic dystrophy type 1 (DM1), only one FDG-PET study used statistical parametric mapping (SPM) showing frontal reduced FDG-uptake. Our aim was to 1) identify the FDG-PET area with the most severe reduced FDG-uptake using SPM8 in a larger group of patients 2) assess potential correlation between CTG-numbers and FDG-PET.

**Methods:**

FDG-PET was performed in 24 patients and compared to 24 controls. Pearson’s correlation was used to analyse correlation.

**Results:**

SPM8 revealed Brodmann area 8 as the area with the most severe reduced FDG-uptake. Weak, although not statistically significant, correlation was observed between CTG-numbers and reduced FDG-uptake in Brodmann area 8.

**Conclusion:**

In DM1, Brodmann area 8 is the area with the most severe reduced FDG-uptake on FDG-PET. Brodmann area 8 reduced FDG-uptake is correlated –although weakly- to CTG-repeat numbers.

## Background

Myotonic dystrophy type 1 (DM1) is caused by an expanded (CTG)n repeat within the noncoding 3’ untranslated region of the myotonic dystrophy protein kinase gene. Cerebral involvement, based on clinical, neuroimaging, and neuropathological evidence, is frequent in DM1 [1–10].

Relatively few reports analysed 18 F-deoxy-glucose positron emission tomography (FDG-PET) in DM1 [1–4]. Only one large study used statistical parametric mapping (SPM), showing bilateral frontotemporal reduced FDG-uptake in the 17 studied patients [1]. In a subset of those 17 patients, a T1-weighted MRI-based partial volume correction was applied. In these patients, bilateral frontal reduced FDG-uptake was observed indicating that frontal reduced FDG-uptake is probably an independent (i.e. not associated with focal brain atrophy) phenomenon in DM1. In their study, no significant correlation was found between FDG-PET metabolism and neuropsychological test results. Correlation between the number of CTG repeats and cerebral metabolism was not analysed directly in that study. One FDG-PET study, using a kinetic modelling to determine the local glucose consumption rate, found that cerebral glucose reduced FDG-uptake was a triplet-size dependent phenomenon. This method, however, requires dynamic PET acquisitions and patient’s blood sampling after 18 F-deoxyglucose injection.

Our aim was to analyse FDG-PET by SPM in a larger group of DM1 patients in order to identify the area with the most severe reduced FDG-uptake and to assess potential correlation between CTG numbers and FDG-uptake. Our study focused on FDG-PET and its relation to CTG-repeats. We did not search for a relationship of these parameters with clinical brain involvement (e.g. cognitive deficit, hypersomnia).

## Methods

### Patients

In our centre, 48 symptomatic and genetically proven adult DM1 patients are in follow-up. Twenty four of these patients accepted to undergo brain FDG-PET. Ethical approval was not needed for our study, since the ethics committee of CHU Nîmes (France) considered the FDG-PET scan as a standard procedure in DM1, if informed consent was given by the patients. All participants gave their informed consent to publish indirect identifiers such as age and gender. All patients gave their informed consent. Patient’s characteristics are summarized in Table 1. Characteristics of included patients were: men/women ratio = 9/15, mean age of 47 years (SD 12.5, range 26–74) at time of brain imaging, mean age of symptom onset of 27 years (range 5–58, including 6 patients with symptom onset before 18 years of age [range 5–14] and 18 patients with adult onset disease), mean disease duration of 20 years (range 6–41), and mean number of 799 CTG repeats (range 83–2000, including 14 patients with CTG <1000 and 10 patients with CTG of ≥1000). None of our patients had congenital DM1. Six out of 24 DM1 patients had associated diabetes (including five patients treated with oral antidiabetic drugs only and one [patient nr 23] with insulin treatment).

**Table 1:** Patient characteristics

Patient Number	Sex	Age symptom onset	Age at FDG-PET	CTG expansion	Diabetes (glucose level)
1	F	58 (late onset)	64	83	No (<120 mg/dl)
2	M	35 (late onset)	74	100	No (<120 mg/dl)
3	F	45 (late onset)	64	150	Yes (120–160 mg/dl)
4	F	32 (late onset)	46	176	No (<120 mg/dl)
5	F	25 (late onset)	41	230	No (<120 mg/dl)
6	F	30 (late onset)	49	330	No (<120 mg/dl)
7	M	26 (late onset)	36	400	No (<120 mg/dl)
8	M	30 (late onset)	50	400	Yes (120–160 mg/dl)
9	F	19 (late onset)	26	400	No (<120 mg/dl)
10	F	30 (late onset)	49	400	No (<120 mg/dl)
11	M	40 (late onset)	58	430	No (<120 mg/dl)
12	F	14 (early onset)	34	660	No (<120 mg/dl)
13	F	13 (early onset)	27	660	No (<120 mg/dl)
14	F	14 (early onset)	31	670	No (<120 mg/dl)
15	F	28 (late onset)	54	850	Yes (120–160 mg/dl)
16	F	39 (late onset)	47	1000	No (<120 mg/dl)
17	M	40 (late onset)	51	1168	Yes (<120 mg/dl)
18	M	24 (late onset)	44	1300	No (<120 mg/dl)c
19	F	28 (late onset)	50	1400	No (<120 mg/dl)
20	M	29 (late onset)	46	1500	No (<120 mg/dl)
21	F	38 (late onset)	58	1560	No (<120 mg/dl)
22	M	5 (early onset)	41	1600	No (<120 mg/dl)
23	M	14 (early onset)	55	1700	Yes (<120 mg/dl)
24	F	6 (early onset)	26	2000	Yes (<120 mg/dl)

Analysis was done for the total of 24 DM1 patients versus 24 controls. For SPM analysis, the control group consisted of 24 subjects (including 10 women and 14 men; mean age 52, SD 10.5, range 32–70) who had neither a history of neurological and psychiatric illness nor abnormalities on neurological examination. FDG-PET scans in controls were performed for study reasons only.

### FDG-PET

All brain FDG-PET scans were done on a PET-CT GEMINI GXL (Philips Medical Systems). After fasting for at least 6 h, blood glucose level was checked and less than 160 mg/dl. The accepted glucose level of 160 mg/dl in our study was relatively high (in contrast with the more commonly used cutoff of 120 mg/dl) because of frequently associated diabetes in DM1 patients. Mean glucose level in our DM1 patients was 103 mg/dl (range 80–159, of whom only 3 patients [i.e. patient nr 3, 8, and 15] had blood glucose levels between 120 and 160 mg/dl). Patients were positioned comfortably in a quiet, dimly lit room before FDG administration and during the uptake phase of FDG (at least 20 min). They received intravenous injection of 185 to 250 MBq (5 to 6.7 mCi: according to the weight) of ^18^F-FDG by a canula inserted 10 min before. They were instructed not to speak, read or be otherwise active. For imaging, patients were in supine position and their head immobilized in a masthead. Imaging started with a CT surview (view angle 90, Kv 120, 30 mAs), then transmission CT scan for attenuation correction was done (120 KV, Mas/slice 200, Pitch 0.563, Rotation 1.5, thickness 3 mm, filter UB, collimation 16 × 1.5, FOV 600); static emission scan started 30 min after injection, in 3-D mode for 20 min, axial field of view 180 mm, 256 × 256 matrix, voxel size 2 mm^3^. PET raw data were corrected for attenuation, random effects and scatter, the reconstruction was then done with a three dimensional row-action maximum likehood algorithm LOR-RAMLA resulted in 90 transaxial slices.

Statistical analysis were done using SPM8 software (Wellcome Department of Imaging Neuroscience, London; http://www.fil.ion.ucl.ac.uk/spm) running on Matlab R2012b version (The Mathworks Inc., USA) running on a Windows XP. Images were realigned and spatially normalized into the MNI (Montreal Neurological Institute, McGill University, Montreal, QC, Canada) standard template given by SPM software using affine and non-linear transformation. Images were reformatted to a final voxel size of 2 × 2 × 2 mm and smoothed using an isotropic Gaussian kernel of 12 × 12 × 12 mm FWHM. A global normalization for voxel count was performed using a proportional scaling. Statistical comparisons between groups were performed on a voxel-by-voxel basis t statistics, using "two-sample t-test" design of the SPM8 generating SPM (t) maps. We investigated brain areas with reduced FDG-uptake, a stringent level of significance of *p* < 0.05 corrected for multiple comparisons (SPM family wise error—FWE) was adopted with an extent threshold of 100 voxels. For visualization the significant voxels were projected onto the 3D rendered brain or a standard MRI template allowing anatomic identification. A MNI to Talairach coordinates conversion was done using Yale Bioimage Suite software (http://www.bioimagesuite.org). On subsequent analyses by lowering *p*-values successively, we searched for the brain area with the most severe reduced FDG-uptake.

### Statistical analyses

Pearson product–moment correlation coefficient was used to analyse potential correlation between the number of CTG repeats and reduced FDG-uptake (by using Z-scores of individual patients). We also compared (using Student’s t-test) uptake between the group of patients with CTG <1000 versus CTG ≥1000 and between the group of patients with early, childhood (age <18) onset versus late, adult (age >18) onset.

The 24 controls were part of a larger control group of 35 cases (age range 32–86). Only the youngest 24 controls were selected in order to avoid possible age-related bias. Difference in age between the 24 DM1 patients and the 24 controls were analyzed by a 2-sample t-test.

In all analyses, when comparing (sub)groups, a threshold of *p* < 0.05 was used to differentiate significant from non-significant differences.

## Results

The age difference between DM1 patients (mean age 47) and controls (mean age 52) was statistically not significant.

**Fig. 1:**
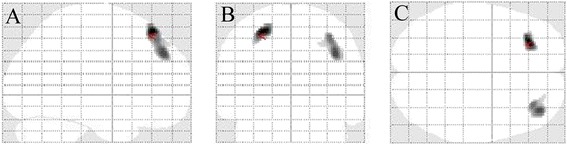
Compared with controls, SPM analysis (**a**, sagittal view; **b**, coronal view, and **c**, axial view) of our 24 DM1 patients showing bilateral reduced FDG-uptake in the lateral part of the frontal lobes, with Brodmann area 8 as the zone with the most severe reduced FDG-uptake

SPM8 analysis of cerebral FDG-PET metabolism in our 24 DM1 patients revealed significant bilateral symmetrical reduced FDG-uptake in the lateral part of the frontal lobes (Fig. 1). The area with the most severe reduced FDG-uptake was Brodmann area 8, associated with slight involvement of Brodmann area 9 on the right side. Deep grey matter structures did not show reduced metabolism. When using an extent threshold of 50 voxels, FDG-PET showed no supplementary areas with reduced FDG-uptake compared to when using an extent threshold of 100 voxels.

The number of CTG repeats was not correlated with FDG-PET metabolism neither for the entire brain nor for the frontal lobes (considered as a whole). Positive -but statistically not significant- correlation was found between the CTG numbers and the metabolism in Brodmann area 8 (especially for the right side, rho = −0.36; rho = −0.22 for the left side).

When comparing patients with <1000 CTG and ≥1000 CTG, patients with ≥1000 CTG showed lower Z-scores in the Brodmann area 8 (median −0.69 for the right and −0.75 for the left Brodmann area 8) than patients with <1000 CTG (median −0.46 for the right and −0.58 for the left Brodmann area 8) although not statistically significant (*p* = 0.28 and *p* = 0.52 for the right and the left side respectively).

When comparing patient with early (childhood) and late (adult) onset DM1, early onset patients showed lower Z-scores (median −0.81 for the right and −0.78 for the left Brodmann area 8) than patients with late onset (median −0.53 for the right and −0.72 for the left Brodmann area 8), without statistically significance (*p* = 0.085 and *p* = 0.5 for the right and the left side respectively).

SPM analyses did not show significant differences between diabetic (*n* = 6) and non-diabetic (*n* = 18) DM1 patients or between patients with blood glucose levels <120 mg/dl (*n* = 21) and patients with blood glucose levels between 120 and 160 mg/dl (*n* = 3).

## Discussion

To the best of our knowledge, this is the largest study analysing FDG-PET by SPM in DM1. We found lateral frontal reduced FDG-uptake as predominant FDG-PET pattern, confirming the data of the only existing study using SPM. The zone with reduced FDG-uptake in our study (although not partial volume corrected) was more restricted than the reported area in the earlier study (using partial volume correction). This might be related to the higher number of patients analyzed and/or due the lack of partial volume correction in our study. The area with the most severe reduced FDG-uptake in our study was identified as Brodmann area 8, associated with slight involvement of the right Brodmann area 9. Although statistically not significant, reduced FDG-uptake was more severe in these brain areas in patients with early, childhood (opposed to late, adult) onset and in patients with ≥1000 (opposed to <1000) CTG.

CTG numbers typically differ between different tissues (e.g. between blood and brain tissue). Therefore, the CTG repeat number obtained by blood sample (like in our study) does not necessarily reflect the number of CTG repeats in the brain (and the brain abnormalities found on FDG-PET possibly related to these repeat numbers). The absence of weak relationship between these blood CTG repeat numbers and (clinical or radiological) brain abnormalities in the earlier reports and our study might be explained in part by this phenomenon.

Interestingly, the area with the most severe reduced FDG-uptake in our DM1 patients was Brodmann area 8, an area involved in (or at least very near to zones implicated in) eye movement control (Brodmann area 8A corresponding to the frontal eye field and area 8B corresponding to the premotor ear-eye field, although the exact localisation of these fields are still under debate). A multitude of oculomotor abnormalities have been reported in DM1 [11–13]. Some of these oculomotor abnormalities are thought to be due to oculomotor muscle dysfunction (weakness and/or myotonia) whereas other abnormalities suggest brain dysfunction. Studies are needed to analyse the relationship between oculomotor deficits (especially those related to dysfunction of the frontal eye field and the premotor ear-eye field) and FDG-PET metabolism.

## Conclusion

In DM1, Brodmann area 8 is the area with the most severe reduced FDG-uptake on FDG-PET. Brodmann area 8 reduced FDG-uptake is weakly correlated to CTG-repeat numbers.

## Abbreviations

DM1, myotonic dystrophy type 1; FDG-PET, 18 F-deoxy-glucose positron emission tomography; SPM, statistical parametric mapping
